# 1-(4-Fluoro­phen­yl)-2-(1*H*-imidazol-1-yl)ethanone

**DOI:** 10.1107/S1600536811026432

**Published:** 2011-07-13

**Authors:** Dong-liang Liu, Chen Li, Guang-yan Yu, Tao Xiao

**Affiliations:** a Department of Applied Chemistry, College of Science, Nanjing University of Technology, Nanjing 210009, People’s Republic of China

## Abstract

In the title compound, C_11_H_9_FN_2_O, the dihedral angle between the rings is 87.50 (4)°. In the crystal, inter­molecular C—H⋯N hydrogen bonds link the mol­ecules in a stacked arrangement along the *c* axis.

## Related literature

For related compounds containing a 2-(1*H*-imidazol-1-yl)-1-phenyl­ethanone fragment, see: Akira *et al.* (1985 ▶); North *et al.* (1968 ▶); Yoshimi *et al.* (2000 ▶); Yuan *et al.* (2007 ▶); Tao *et al.* (2007 ▶). For standard bond lengths, see: Allen *et al.* (1987 ▶).
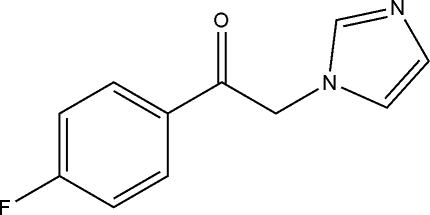


## Experimental

### 

#### Crystal data

C_11_H_9_FN_2_O
*M* *_r_* = 204.20
Orthorhombic, 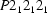

*a* = 8.6730 (17) Å
*b* = 10.132 (2) Å
*c* = 11.088 (2) Å
*V* = 974.4 (3) Å^3^
*Z* = 4
Mo *K*α radiation
μ = 0.11 mm^−1^
*T* = 293 K
0.30 × 0.20 × 0.10 mm

#### Data collection

Enraf–Nonius CAD-4 diffractometer
Absorption correction: ψ scan (North *et al.*, 1968 ▶) *T*_min_ = 0.969, *T*_max_ = 0.990
3790 measured reflections
1052 independent reflections
778 reflections with *I* > 2σ(*I*)
*R*_int_ = 0.045
3 standard reflections every 200 reflections  intensity decay: 1%

#### Refinement

*R*[*F* ^2^ > 2σ(*F* ^2^)] = 0.037
*wR*(*F* ^2^) = 0.093
*S* = 1.01
1052 reflections
137 parameters
H-atom parameters constrained
Δρ_max_ = 0.10 e Å^−3^
Δρ_min_ = −0.09 e Å^−3^

### 

Data collection: *CAD-4 EXPRESS* (Enraf–Nonius, 1994) ▶; cell refinement: *CAD-4 EXPRESS* ▶; data reduction: *XCAD4* (Harms & Wocadlo,1995 ▶); program(s) used to solve structure: *SHELXS97* (Sheldrick, 2008 ▶); program(s) used to refine structure: *SHELXL97* (Sheldrick, 2008 ▶); molecular graphics: *PLATON* (Spek, 2009) ▶; software used to prepare material for publication: *SHELXTL* (Sheldrick, 2008 ▶).

## Supplementary Material

Crystal structure: contains datablock(s) I, global. DOI: 10.1107/S1600536811026432/zq2112sup1.cif

Structure factors: contains datablock(s) I. DOI: 10.1107/S1600536811026432/zq2112Isup3.hkl

Supplementary material file. DOI: 10.1107/S1600536811026432/zq2112Isup3.cml

Additional supplementary materials:  crystallographic information; 3D view; checkCIF report

## Figures and Tables

**Table 1:** Hydrogen-bond geometry (Å, °)

*D*—H⋯*A*	*D*—H	H⋯*A*	*D*⋯*A*	*D*—H⋯*A*
C8—H8*A*⋯N2^i^	0.97	2.51	3.454 (4)	164

Symmetry code: (i) 
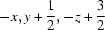
.

**Fig. 1.**
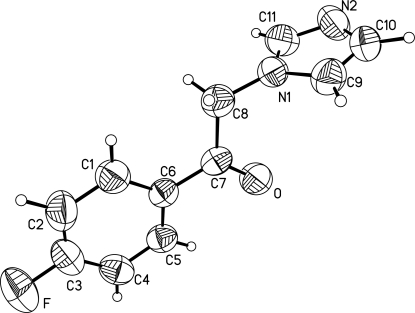
The molecular structure of the title compound with displacement ellipsoids drawn at the 50% probability level.

**Fig. 2.**
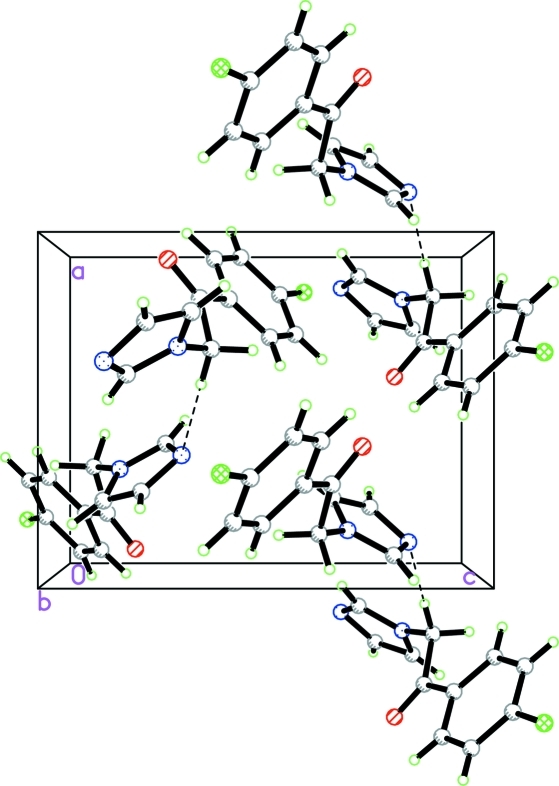
A packing diagram of the title compound. Hydrogen bonds are shown as dashed lines.

## supplementary crystallographic information

### Comment

The title compound, C_11_H_9_O_1_N_2_F_1_, is the key intermediate in the synthesis of a new kind of antifungal drug (Akira *et al.*, 1985; Yoshimi *et al.*, 2000). The crystal structure determination has been carried out in order to elucidate the molecular conformation (Fig. 1).

In the crystal structure, the bond lengths and angles of the title compound are within normal ranges (Allen *et al.*, 1987). The phenyl and imidazole rings are planar (rms deviations of 0.0038 and 0.0036, respectively) and almost perpendicular to each other. The dihedral angle between the mean planes is 87.50 (4)°. In the crystal structure, intermolecular C—H···N hydrogen bonds (Table 1) link the molecules in a stacked arrangement along the *a* axis (Fig. 2).

### Experimental

Sodium hydride (4.8 g, 120 mmol) was suspended in dimethylformamide (DMF, 30 ml). Imidazole (6.8 g, 120 mmol) dissolved in DMF (30 ml) was slowly added dropwise at 273 K, and reacted atroom temperature for 30 min. 2-chloro-1-(4-fluorophenyl)ethanone (15.48 g, 90 mmol) dissolved in DMF (30 ml) was then slowly added dropwise, and reacted at room temperature for 4 h. The mixture was placed in ice-water (300 ml), and 1 mol hydrochloric acid (50 ml) was then added. After filtration, the filtrate was neutralized with sodium bicarbonate to pH = 6, and a yellow deposit was obtained (m.p. 423–424 K). Crystals suitable for X-ray analysis were obtained by dissolving the crude product (1.0 g) in ethanol (30 ml) and then allowing the solution to evaporate slowly at room temperature for about 7 d.

### Refinement

In the absence of significant anomalous scattering effects, all Friedel pairs were merged. H atoms were positioned geometrically, with C—H = 0.93 and 0.97 Å for aromatic and methylene H atoms, respectively, and constrained to ride on their parent atoms with *U*_iso_(H) = 1.2*U*_eq_(C).

### Crystal data

**Table:**

C_11_H_9_FN_2_O	*D*_x_ = 1.392 Mg m^−^^3^
*M**_r_* = 204.20	Melting point: 423 K
Orthorhombic, *P*2_1_2_1_2_1_	Mo *K*α radiation, λ = 0.71073 Å
Hall symbol: P 2ac 2ab	Cell parameters from 25 reflections
*a* = 8.6730 (17) Å	θ = 9–13°
*b* = 10.132 (2) Å	µ = 0.11 mm^−^^1^
*c* = 11.088 (2) Å	*T* = 293 K
*V* = 974.4 (3) Å^3^	Block, yellow
*Z* = 4	0.30 × 0.20 × 0.10 mm
*F*(000) = 424	

### Data collection

**Table:**

Enraf–Nonius CAD-4 diffractometer	778 reflections with *I* > 2σ(*I*)
Radiation source: fine-focus sealed tube	*R*_int_ = 0.045
graphite	θ_max_ = 25.4°, θ_min_ = 2.7°
ω/2θ scans	*h* = 0→10
Absorption correction: ψ scan (North *et al.*, 1968)	*k* = −12→12
*T*_min_ = 0.969, *T*_max_ = 0.990	*l* = −13→13
3790 measured reflections	3 standard reflections every 200 reflections
1052 independent reflections	intensity decay: 1%

### Refinement

**Table:**

Refinement on *F*^2^	Secondary atom site location: difference Fourier map
Least-squares matrix: full	Hydrogen site location: inferred from neighbouring sites
*R*[*F*^2^ > 2σ(*F*^2^)] = 0.037	H-atom parameters constrained
*wR*(*F*^2^) = 0.093	*w* = 1/[σ^2^(*F*_o_^2^) + (0.0484*P*)^2^] where *P* = (*F*_o_^2^ + 2*F*_c_^2^)/3
*S* = 1.01	(Δ/σ)_max_ < 0.001
1052 reflections	Δρ_max_ = 0.10 e Å^−^^3^
137 parameters	Δρ_min_ = −0.09 e Å^−^^3^
0 restraints	Extinction correction: *SHELXL97* (Sheldrick, 2008), Fc^*^=kFc[1+0.001xFc^2^λ^3^/sin(2θ)]^-1/4^
Primary atom site location: structure-invariant direct methods	Extinction coefficient: 0.034 (5)

### Special details

**Table:**

Geometry. All e.s.d.'s (except the e.s.d. in the dihedral angle between two l.s. planes) are estimated using the full covariance matrix. The cell e.s.d.'s are taken into account individually in the estimation of e.s.d.'s in distances, angles and torsion angles; correlations between e.s.d.'s in cell parameters are only used when they are defined by crystal symmetry. An approximate (isotropic) treatment of cell e.s.d.'s is used for estimating e.s.d.'s involving l.s. planes.
Refinement. Refinement of *F*^2^ against ALL reflections. The weighted *R*-factor *wR* and goodness of fit *S* are based on *F*^2^, conventional *R*-factors *R* are based on *F*, with *F* set to zero for negative *F*^2^. The threshold expression of *F*^2^ > σ(*F*^2^) is used only for calculating *R*-factors(gt) *etc*. and is not relevant to the choice of reflections for refinement. *R*-factors based on *F*^2^ are statistically about twice as large as those based on *F*, and *R*- factors based on ALL data will be even larger.

### Fractional atomic coordinates and isotropic or equivalent isotropic displacement parameters (Å^2^)

**Table:**

	*x*	*y*	*z*	*U*_iso_*/*U*_eq_	
F	0.3432 (3)	0.73232 (18)	0.41347 (18)	0.1052 (8)	
O	0.4019 (3)	0.2049 (2)	0.7042 (2)	0.0838 (8)	
N1	0.1657 (3)	0.0314 (2)	0.6780 (2)	0.0556 (6)	
C1	0.2015 (4)	0.4109 (3)	0.4913 (3)	0.0657 (9)	
H1A	0.1223	0.3538	0.4695	0.079*	
C2	0.2138 (4)	0.5322 (3)	0.4355 (3)	0.0760 (10)	
H2A	0.1439	0.5574	0.3763	0.091*	
N2	0.1233 (3)	−0.1189 (3)	0.8187 (3)	0.0798 (9)	
C3	0.3297 (5)	0.6133 (3)	0.4687 (3)	0.0708 (9)	
C4	0.4345 (4)	0.5823 (3)	0.5561 (3)	0.0671 (9)	
H4A	0.5123	0.6409	0.5776	0.081*	
C5	0.4208 (3)	0.4608 (3)	0.6112 (2)	0.0568 (8)	
H5A	0.4906	0.4372	0.6711	0.068*	
C6	0.3053 (3)	0.3735 (2)	0.5794 (2)	0.0496 (7)	
C7	0.3005 (3)	0.2428 (3)	0.6385 (2)	0.0535 (7)	
C8	0.1625 (3)	0.1556 (3)	0.6133 (3)	0.0623 (8)	
H8A	0.0694	0.2031	0.6351	0.075*	
H8B	0.1579	0.1376	0.5275	0.075*	
C9	0.2500 (4)	−0.0773 (3)	0.6483 (3)	0.0689 (9)	
H9A	0.3144	−0.0871	0.5818	0.083*	
C10	0.2215 (4)	−0.1673 (3)	0.7341 (3)	0.0726 (9)	
H10A	0.2634	−0.2517	0.7355	0.087*	
C11	0.0935 (4)	0.0013 (3)	0.7805 (3)	0.0702 (9)	
H11A	0.0285	0.0594	0.8209	0.084*	

### Atomic displacement parameters (Å^2^)

**Table:**

	*U*^11^	*U*^22^	*U*^33^	*U*^12^	*U*^13^	*U*^23^
F	0.143 (2)	0.0728 (12)	0.1000 (14)	−0.0026 (14)	0.0088 (16)	0.0255 (11)
O	0.0624 (13)	0.0863 (16)	0.1027 (17)	−0.0131 (12)	−0.0320 (15)	0.0296 (14)
N1	0.0520 (14)	0.0485 (13)	0.0663 (15)	0.0000 (12)	−0.0001 (13)	−0.0063 (12)
C1	0.064 (2)	0.071 (2)	0.0621 (18)	−0.0075 (18)	−0.0060 (17)	0.0042 (16)
C2	0.081 (3)	0.077 (2)	0.069 (2)	0.006 (2)	−0.013 (2)	0.0144 (18)
N2	0.087 (2)	0.0536 (16)	0.099 (2)	−0.0046 (15)	0.0121 (19)	0.0027 (15)
C3	0.092 (3)	0.0574 (18)	0.063 (2)	0.004 (2)	0.015 (2)	0.0078 (16)
C4	0.073 (2)	0.0598 (18)	0.0681 (19)	−0.0104 (17)	0.009 (2)	−0.0072 (16)
C5	0.0529 (17)	0.0658 (19)	0.0518 (17)	−0.0018 (15)	0.0016 (15)	−0.0025 (14)
C6	0.0467 (16)	0.0559 (16)	0.0463 (15)	0.0017 (14)	0.0002 (13)	−0.0028 (12)
C7	0.0445 (15)	0.0635 (17)	0.0524 (16)	0.0015 (14)	0.0024 (14)	−0.0041 (14)
C8	0.0580 (18)	0.0609 (18)	0.0681 (19)	−0.0017 (15)	−0.0059 (17)	−0.0028 (15)
C9	0.0601 (18)	0.0654 (18)	0.081 (2)	0.0062 (17)	0.0065 (18)	−0.0114 (18)
C10	0.066 (2)	0.0521 (17)	0.100 (2)	0.0043 (16)	0.001 (2)	−0.0078 (19)
C11	0.0656 (19)	0.064 (2)	0.080 (2)	−0.0029 (18)	0.0139 (19)	−0.0142 (18)

### Geometric parameters (Å, °)

**Table:**

F—C3	1.358 (3)	C4—C5	1.379 (4)
O—C7	1.205 (3)	C4—H4A	0.9300
N1—C11	1.333 (3)	C5—C6	1.382 (4)
N1—C9	1.363 (3)	C5—H5A	0.9300
N1—C8	1.449 (3)	C6—C7	1.478 (4)
C1—C2	1.380 (4)	C7—C8	1.514 (4)
C1—C6	1.381 (4)	C8—H8A	0.9700
C1—H1A	0.9300	C8—H8B	0.9700
C2—C3	1.350 (5)	C9—C10	1.341 (4)
C2—H2A	0.9300	C9—H9A	0.9300
N2—C11	1.315 (4)	C10—H10A	0.9300
N2—C10	1.359 (4)	C11—H11A	0.9300
C3—C4	1.365 (5)		
			
C11—N1—C9	105.9 (3)	C1—C6—C7	122.7 (3)
C11—N1—C8	127.7 (3)	C5—C6—C7	118.7 (3)
C9—N1—C8	126.3 (3)	O—C7—C6	122.2 (3)
C2—C1—C6	120.7 (3)	O—C7—C8	120.2 (3)
C2—C1—H1A	119.6	C6—C7—C8	117.6 (3)
C6—C1—H1A	119.6	N1—C8—C7	113.6 (2)
C3—C2—C1	118.5 (3)	N1—C8—H8A	108.8
C3—C2—H2A	120.7	C7—C8—H8A	108.8
C1—C2—H2A	120.7	N1—C8—H8B	108.8
C11—N2—C10	103.6 (3)	C7—C8—H8B	108.8
C2—C3—F	118.8 (3)	H8A—C8—H8B	107.7
C2—C3—C4	123.3 (3)	C10—C9—N1	106.2 (3)
F—C3—C4	117.9 (3)	C10—C9—H9A	126.9
C3—C4—C5	117.6 (3)	N1—C9—H9A	126.9
C3—C4—H4A	121.2	C9—C10—N2	111.1 (3)
C5—C4—H4A	121.2	C9—C10—H10A	124.4
C4—C5—C6	121.4 (3)	N2—C10—H10A	124.4
C4—C5—H5A	119.3	N2—C11—N1	113.2 (3)
C6—C5—H5A	119.3	N2—C11—H11A	123.4
C1—C6—C5	118.5 (3)	N1—C11—H11A	123.4
			
C6—C1—C2—C3	0.0 (5)	C5—C6—C7—C8	172.8 (2)
C1—C2—C3—F	179.5 (3)	C11—N1—C8—C7	98.2 (3)
C1—C2—C3—C4	−0.9 (6)	C9—N1—C8—C7	−78.6 (4)
C2—C3—C4—C5	0.8 (5)	O—C7—C8—N1	2.1 (4)
F—C3—C4—C5	−179.5 (3)	C6—C7—C8—N1	−178.2 (2)
C3—C4—C5—C6	0.0 (4)	C11—N1—C9—C10	0.9 (3)
C2—C1—C6—C5	0.8 (4)	C8—N1—C9—C10	178.3 (3)
C2—C1—C6—C7	−177.6 (3)	N1—C9—C10—N2	−0.9 (4)
C4—C5—C6—C1	−0.8 (4)	C11—N2—C10—C9	0.5 (4)
C4—C5—C6—C7	177.6 (3)	C10—N2—C11—N1	0.1 (4)
C1—C6—C7—O	170.8 (3)	C9—N1—C11—N2	−0.6 (4)
C5—C6—C7—O	−7.6 (4)	C8—N1—C11—N2	−177.9 (3)
C1—C6—C7—C8	−8.9 (4)		

### Hydrogen-bond geometry (Å, °)

**Table:**

*D*—H···*A*	*D*—H	H···*A*	*D*···*A*	*D*—H···*A*
C8—H8A···N2^i^	0.97	2.51	3.454 (4)	164

Symmetry codes: (i) −*x*, *y*+1/2, −*z*+3/2.
